# Pleiohomeotic Interacts with the Core Transcription Elongation Factor Spt5 to Regulate Gene Expression in *Drosophila*

**DOI:** 10.1371/journal.pone.0070184

**Published:** 2013-07-22

**Authors:** Robert Harvey, Eugene Schuster, Barbara H. Jennings

**Affiliations:** 1 Transcriptional Regulation Group, UCL Cancer Institute, University College London, London, United Kingdom; 2 Department of Genetics, Evolution and Environment, University College London, London, United Kingdom; Cardiff University, United Kingdom

## Abstract

The early elongation checkpoint regulated by Positive Transcription Elongation Factor b (P-TEFb) is a critical control point for the expression of many genes. Spt5 interacts directly with RNA polymerase II and has an essential role in establishing this checkpoint, and also for further transcript elongation. Here we demonstrate that *Drosophila* Spt5 interacts both physically and genetically with the Polycomb Group (PcG) protein Pleiohomeotic (Pho), and the majority of Pho binding sites overlap with Spt5 binding sites across the genome in S2 cells. Our results indicate that Pho can interact with Spt5 to regulate transcription elongation in a gene specific manner.

## Introduction

The regulation of the early phase of transcriptional elongation is used to control the expression of many genes. When this process fails it leads to death or severe defects during development and contributes to cancer pathogenesis in adult animals [1].

Once transcription has been initiated by recruitment of the pre-initiation complex (PIC), RNA polymerase II (RNAP II) transcribes 20–40 base pairs but then must pass through a checkpoint regulated by Positive Transcription Elongation Factor b (P-TEFb) to produce full-length transcripts (recently reviewed in [2], [3], [4]). Two protein complexes act together to inhibit transcript elongation beyond ∼25–40 nucleotides after initiation. One of these is made up of the Spt5 and Spt4 proteins and is sometimes referred to as “DSIF” [5], [6], and the other, Negative Elongation Factor (NELF), contains four subunits (NELF-A, NELF-B, NELF-C/D, NELF-E; [7]). For further elongation to occur, P-TEFb must phosphorylate specific residues in NELF, Spt5, and RNAP II. This induces the dissociation of NELF from the polymerase complex, the switch in Spt5 from being a negative to positive regulator of transcription, and production of the full-length transcript by RNAP II. Spt5 tracks along with the RNAP II elongation complex until transcription termination.

Spt5 is required to establish promoter proximal polymerase pausing at the P-TEFb checkpoint, however, it is essential for productive transcription from all genes. Spt5 is conserved across the three domains of life [Eukaryotes, Archaea and Bacteria (NusG)] and is recruited by RNA polymerases I, II and III [5]. Recent structural studies have shown that the NGN domain of Spt5 sits over the DNA and RNA bound in the active site of RNA polymerases, where it can directly control the rate of transcript elongation [8], [9].

It is well established that the P-TEFb checkpoint is a key point of regulation for many genes. However, the factors that determine which genes are subject to rate-limiting regulation at the P-TEFb checkpoint are largely unknown, as is how they interact with the RNAP II elongation complex to establish promoter proximal pausing.

Missense mutations in *Spt5* that give rise to specific developmental defects have been isolated in zebrafish and *Drosophila* [10], [11] providing evidence that Spt5 activity is responsive to contextual factors controlling gene expression. Zebrafish homozygous for the *Spt5^foggy[m806]^* allele develop quite normally, however they do exhibit a distinctive neural phenotype (excess dopaminergic neurons and fewer serotonergic neurons) and eventually die of vascular defects thought to be a secondary consequence of abnormal neuronal function [10]. Meanwhile, *Drosophila* embryos derived from maternal germline clones homozygous for the *Spt5^W049^* mutation (thus, all protein in the embryo prior to the onset of zygotic transcription is mutant), exhibit segmentation defects stemming from aberrant expression of *even-skipped* (*eve*) and *runt* (*run*). The effects of *Spt5^W049^* are gene-specific, (gap gene and *hairy* expression are normal in *Spt5^W049^* germline clones) and appear to be enhancer-specific for *eve* expression [11]. The single amino acid substitutions found in the Foggy and W049 mutant proteins map close together in the C-terminal region of Spt5, which is conserved in higher metazoans including *Drosophila,* but not found in yeast or *C. elegans*. This region is distinct from the domain in Spt5 that is subject to phosphorylation by P-TEFb, which is sometimes referred to as the Spt5 CTR or CTD domain. Thus to avoid confusion, we will refer to the extreme C-terminal domain of Spt5 found in higher metazoans as the Developmental Domain (DD).

In this study we characterise an interaction between Spt5 and the transcription factor Pleiohomeotic (Pho) that we uncovered using the yeast 2-hybrid assay. We demonstrate that Spt5 and Pho act together in vivo during adult maturation and PcG repression, and that the majority of sites bound by Pho in the genome co-localize to Spt5 and NELF binding sites.

## Results

### Spt5 Interacts with Pho

We performed a yeast 2-hybrid screen using the C-terminal 153 amino acids of *Drosophila* Spt5 as bait to identify factors that interact with the DD. In frame fragments of Pho were recovered from the screen multiple times and did not retest as false positives. Pho is an ortholog of mammalian Ying Yang 1 (YY1) and like Spt5, is a ubiquitously expressed nuclear protein that has been implicated in both transcriptional activation and repression [12], [13].

**Figure 1:**
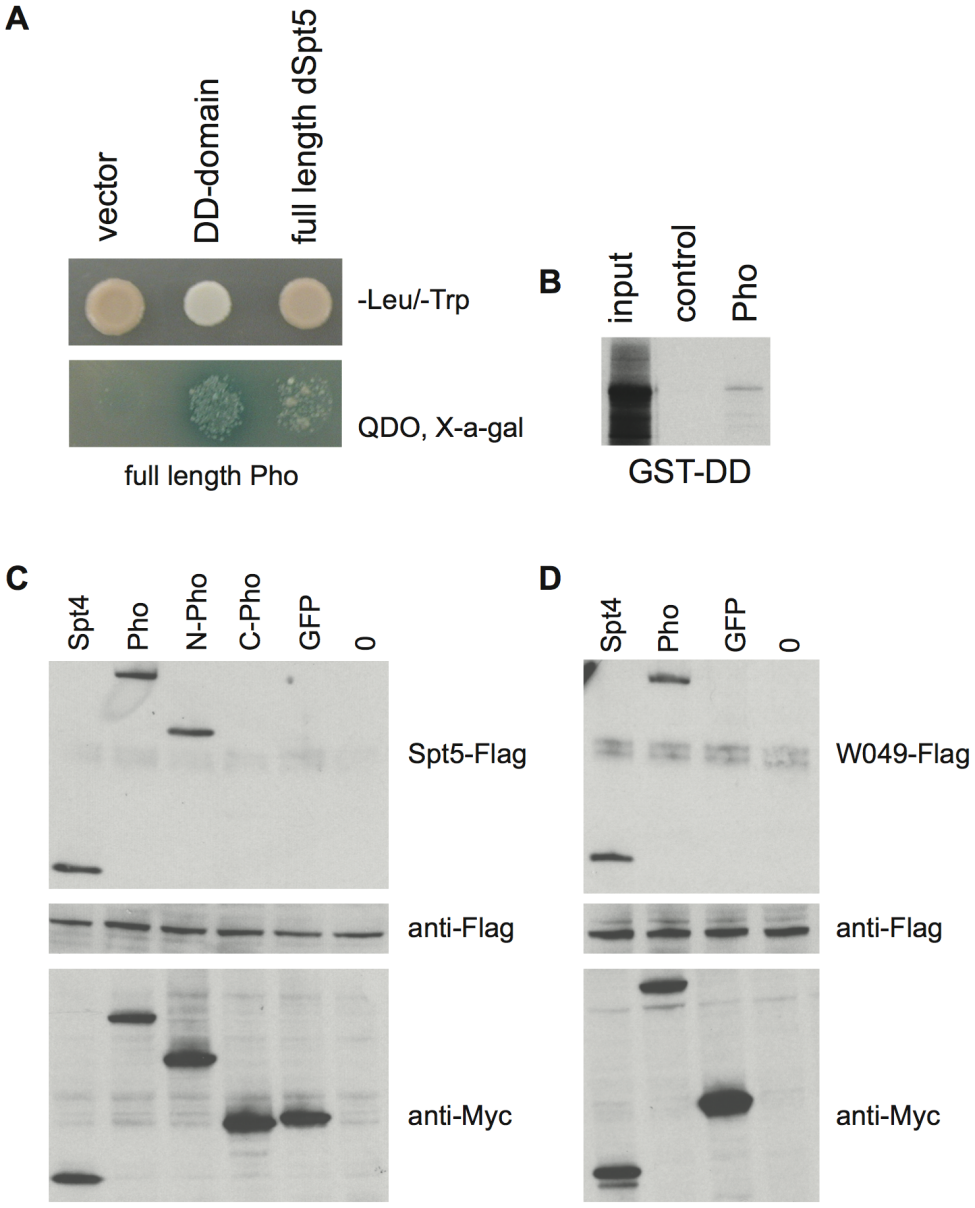
Pho physically interacts with Spt5. A) Yeast 2-hybid assay showing binding of full length Pho with the Spt5 DD domain and full length Spt5. Vector (pGBKT7) containing no insert was used as a control to demonstrate that Pho does not activate reporter gene expression in the absence of Spt5. B) Pho binds to immobilized GST-DD. Ten percent of the input Pho is run in left lane, immobilized GST in middle lane incubated with Pho as negative control. C) Western blots of co-immunoprecipitation (co-IP) assays from S2 cell extracts of Flag-tagged Spt5 with Myc-Spt4 (positive control), Myc-Pho, Myc-N-Pho (amino acids 1–351), Myc-C-Pho (351–520), Myc-GFP (negative control) and no protein. D) Western blots of co-IP assays from S2 cell extracts of Flag-tagged W049 variant of Spt5 with Myc-Spt4 (positive control), Myc-Pho, Myc-GFP (negative control) and no protein.

Full length Spt5 interacts with full length Pho in the yeast 2-hybrid assay (Figure 1A) and the DD also interacted weakly but specifically with Pho in GST pull down assays (Figure 1B). The interaction between full length Spt5 and Pho was further validated by expressing tagged proteins in *Drosophila* S2 cells and performing co-immunoprecipitation assays (Figure 1C). We mapped the interaction with Spt5 to the N-terminal 351-amino acids of Pho using co-immunoprecipitation of tagged proteins (Figure 1C). Sequences within this region have previously been shown to interact with Polycomb (Pc), Polyhomeotic (Ph) and the Brahma (BRM) complex [14]. The C-terminal region of Pho (remaining 169 amino acids), which does not interact with Spt5, contains the 4 zinc finger motif (C2H2-like) that is highly conserved with human YY1 and has been shown to bind DNA [12].

The DD carrying the *W049* (G994D) mutation is able to interact with Pho in the yeast 2-hybrid and GST pull down assays, and full length W049 protein interacts with Pho in the yeast 2-hybrid and co-immunoprecipitation assays (Figure 1D and data not shown). Thus a failure of the interaction between Spt5 and Pho is not likely to explain the phenotypes observed in *Spt5^W049^* mutants. The *W049* mutation may be affecting the ability of Spt5 to interact with other as yet unidentified factors.

### Spt5 Contributes to Pho Mediated Repression of PcG Targets in vivo

We looked for genetic interactions between mutant alleles of *Spt5* and *pho* to assess if they function together in vivo. *pho^cv^* is a hypomorphic allele that is homozygous viable but male sterile [12]. Homozygous *pho^cv^* males exhibit the classic polycomb phenotype of ectopic sex combs on the middle (mesothoracic) and rear (metathoracic) legs due to de-repression of the *Sex combs reduced* (*Scr*) gene [15]. Multiple crosses were done in parallel in uncrowded vials (∼3–6 females and ∼2–4 males) at 25°C. Siblings were scored to reduce effects caused by genetic background and environment.

**Figure 2:**
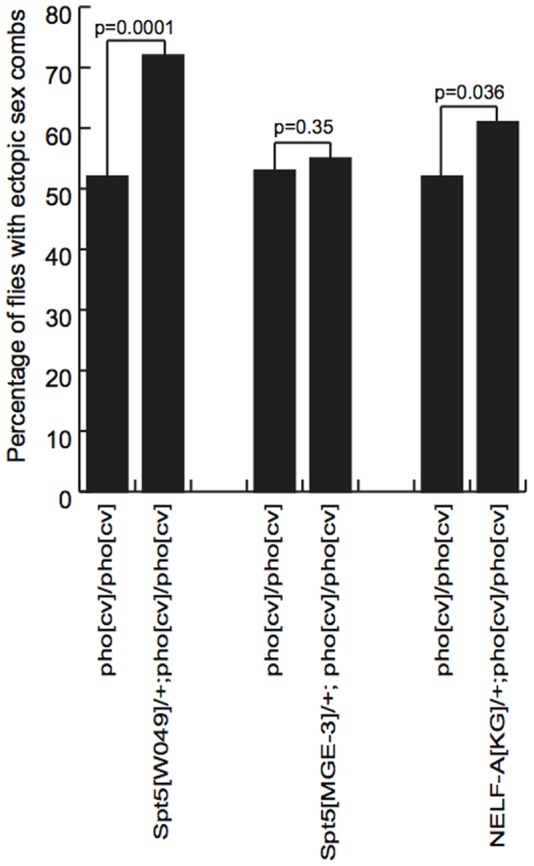
Modification of the extra sex combs phenotype of *pho^cv^/pho^cv^* mutants by *Spt5* and *NELF* mutant alleles. A chart representing the frequency of ectopic sex combs in *pho^cv^/pho^cv^* mutants and siblings heterozygous for *Spt5^W049^*, *Spt5^MGE^* ^−*3*^ or *NELF-A^KG^* over wild-type chromosomes. p values from two proportion z-tests are shown.

We counted the number of flies carrying ectopic sex combs in homozygous *pho^cv^* males and homozygous *pho^cv^* males heterozygous for *Spt5* alleles to determine if *Spt5* interacts with *pho* during PcG repression in vivo. A two-proportion hypothesis test was applied to determine the significance of any differences observed in these frequencies. There was no significant increase in the frequency of ectopic sex combs observed in *pho^cv^*/*pho^cv^* males that are heterozygous for a null allele of Spt5 (*Spt5^MGE^* ^−*3*^) [16], indicating that halving the dose of Spt5 does not compromise residual Pho activity (Figure 2 and Table 1). This is perhaps not unexpected as Spt5 is expressed at moderate or moderately high levels during larval development [17] and *Spt5^MGE^* ^−*3*^/+ flies appear wild-type. However, *Spt5^W049^/+; pho^cv^*/*pho^cv^* males did show a significant increase in the number of ectopic sex combs, revealing that the *W049* variant protein can disrupt the repressive activity of Pho in vivo (Figure 2 and Table 1). We also observed a small but significant increase in the number of ectopic sex combs in *pho^cv^* males heterozygous for *NELF-A^[KG]^* [18] (Figure 2, Table 1).

**Table 1:** Genetic Interactions between *Spt5*, *NELF-A* alleles and *pho^cv^.*

Genotype	Total (n)	Wing Phenotype	Ectopic sex combs
*wild-type*	233	7 (3%)	0
*Spt5[W049]/+*	191	5 (2.6%)	0
*pho[cv]/pho[cv]*	194	19 (9.8%)	102 (53%)
*Spt5[W049]/+; pho[cv]/pho[cv]*	149	45 (30%)	108 (72%)
*Spt5[MGE-3]/+*	170	1 (0.6%)	0
*pho[cv]/pho[cv]*	184	21 (11%)	102 (55%)
*Spt5[MGE-3]/+; pho[cv]/pho[cv]*	198	56 (28%)	106 (54%)
*NELF-A[KG]/+*	166	17 (10%)	0
*pho[cv]/pho[cv]*	175	22 (12%)	92 (53%)
*NELF-A[KG]/+; pho[cv]/pho[cv]*	288	61 (21%)	176 (61%)

The W049 protein has a significantly reduced repressive activity on transcription in vitro, and in some contexts in vivo [11]. W049 allows RNAP II to continue transcribing through the P-TEFb checkpoint in the presence of a P-TEFb inhibitor [5,6-dichloro-1-β-D-ribofuranosylbenzimidazole (DRB)] in nuclear extracts [11]. Thus, the presence of W049 protein has the potential to interfere with repression dependent on the P-TEFb checkpoint in heterozygous flies. *Spt5^W049^/+*flies resemble wild-type, so this effect is only apparent when the function of other factors involved is compromised.

Kwong et al., 2008 observed an enrichment of Pho binding just downstream of the start of transcription of *Scr* in T3 imaginal discs around the predicted site of the P-TEFb checkpoint [19]. Furthermore, the additional ectopic sex combs observed in *NELF-A^KG^/+; pho^cv^*/*pho^cv^* flies is consistent with the model that inhibition of this checkpoint is critical for Pho-mediated PcG repression of *Scr*. Thus, we propose a model in which Pho acts together with Spt5 and NELF to prevent RNAP II transcribing through the P-TEFb checkpoint to maintain PcG repression.

### *Spt5* Genetically Interacts with *pho* during Wing Maturation

**Figure 3:**
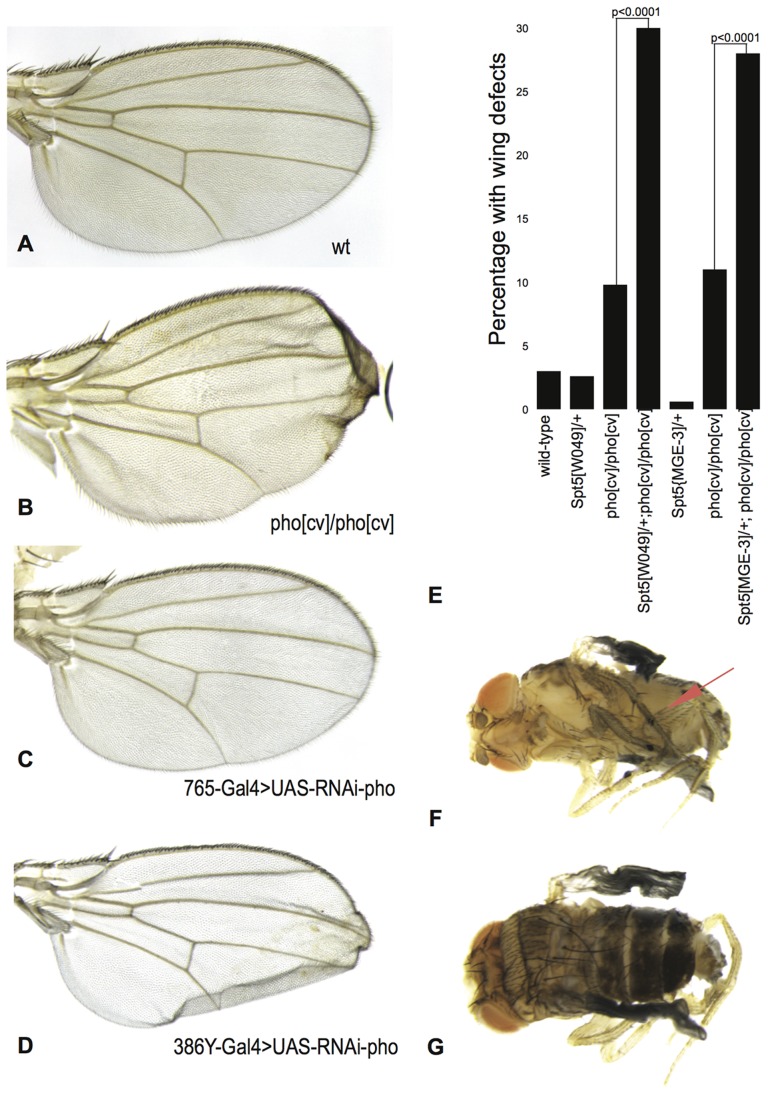
Pho and Spt5 function together in wing maturation. A wing inflation phenotype is observed in approximately 10% of *pho^cv^/pho^cv^* B) and 51% of *386Y-Gal4>UAS-RNAi pho* males (n = 136), but not in *765-Gal4>UAS-RNAi-pho*. E) Percentage of flies of indicated genotypes displaying wing inflation phenotypes. F) Ventral view of *da-Gal4>UAS-RNAi-pho* male, red arrow points to ectopic sex comb on middle (mesothoracic) leg. G) Dorsal view of *da-Gal4>UAS-RNAi-pho* male displaying homeotic transformations in the abdominal segments.

While assessing the various genotypes for Polycomb phenotypes we noticed that approximately 10% of *pho^cv^* homozygotes exhibit a phenotype resulting from aberrant wing inflation and deflation during hatching from the pupal case (eclosion). Introduction of a single copy of *Spt5^W049^* or *Spt5^MGE^* ^−*3*^ into this background increased the frequency to 30% and 28% respectively, demonstrating a significant genetic interaction between the *pho* and *Spt5* loci (Figure 3 and Table 1). All elements of the wing (veins, bristles, and hairs) are present and normal in *pho^cv^* homozygotes, but affected wings were noticeably ruffled along the posterior edge and had regions where the dorsal and ventral surfaces were coming apart. The extent of this phenotype was variable, with some wings also being folded and/or containing small blisters.

A wing inflation phenotype has not previously been described for *pho^c^* ^v^, however the phenotypes of escaper flies homozygous for stronger *pho* alleles support a role for *pho* in wing development. Flies homozygous for *pho^b^* allele [12] die as pharate adults; they make it all the way through development on the maternally supplied Pho, but fail to eclose.

Similarly, expression of *UAS-RNAi-pho* driven ubiquitously throughout development by *da-GAL4* is generally lethal at 18°C, with flies dying as pharate adults. The vast majority of escapers that hatch are unable to fully inflate their wings and remain pale and juvenile looking (91%; n = 67) in addition to having the phenotypes previously described for *pho* mutants including ectopic sex combs and partial homeotic transformations of abdominal segments (Figure 3F and 3G, [20]). Driving ubiquitous expression of *UAS-RNAi-pho* recapitulates the phenotype of strong *pho* alleles in vivo.

**Figure 4:**
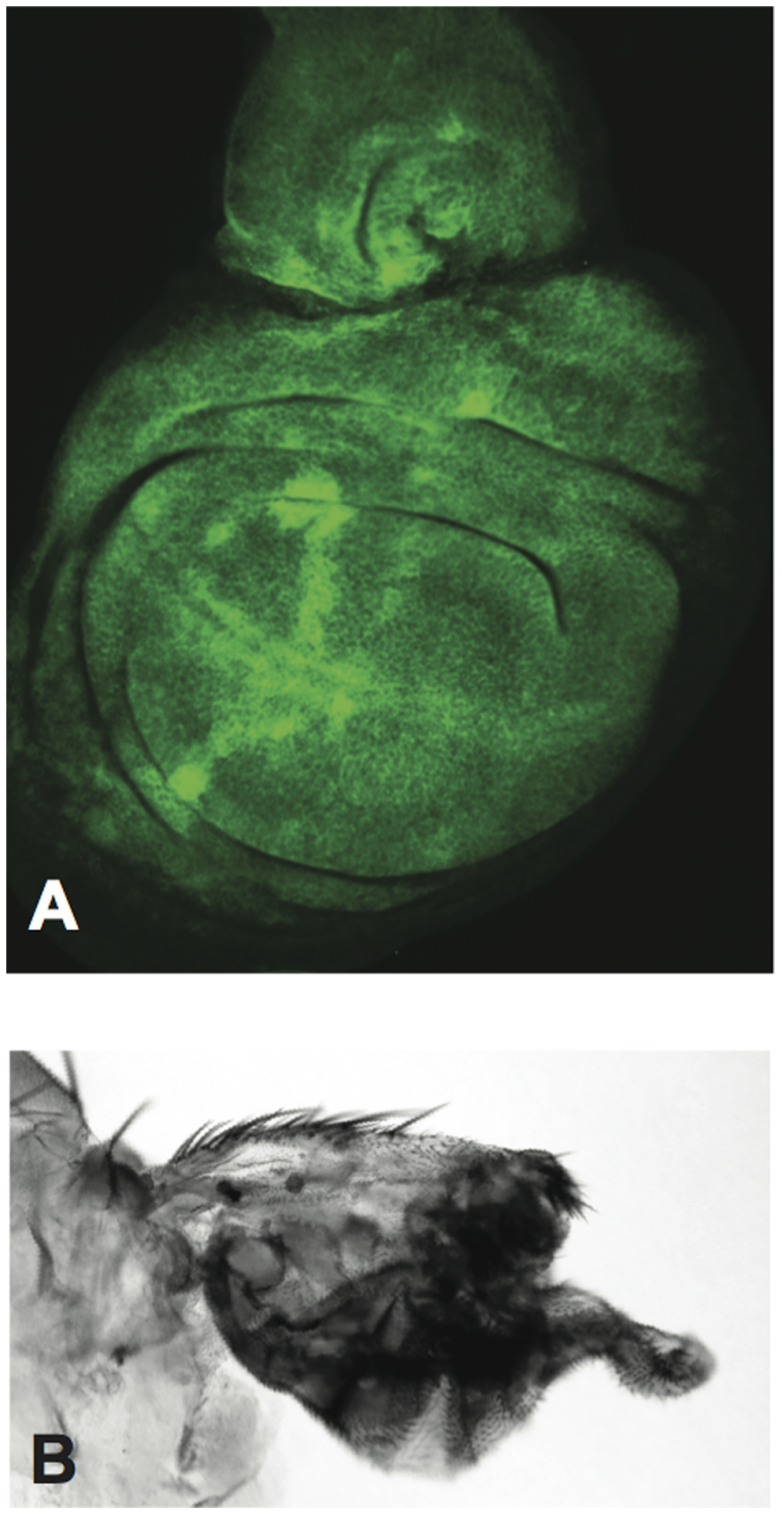
Depletion of Spt5 leads to cell death in vivo. A) Homozygous clones of the *Spt5^MGE^* null allele are not viable. Attempts were made to make clones of homozygous *Spt5^MGE^* cells using the FLP/FRT technique [61]. Third instar imaginal wing disk (anterior to the left and dorsal to the top) stained for GFP. All cells stain green and are thus either heterozygous or homozygous (bright green) for the *FRT42B, GFP* chromosome; loss of GFP would mark clones of homozygous *FRT42B, Spt5^MGE^.* Similarly, when we induced homozygous germ-line clones of *Spt5^MGE^* in females using the FLP/FRT/ovoD technique [62], they did not lay any eggs indicating that homozygous *Spt5^MGE^* clones are cell lethal (data not shown). B) Residual wing stub from fly expressing *765-Gal4>UAS-RNAi-Spt5* at 18°C the portion of the wing expressing *765-Gal4* does not develop as there is a deficit of cells consistent with expression of *UAS-RNAi-Spt5* being lethal to cells.

There are no obvious wing defects when *765-Gal4* drives *UAS-RNAi-pho* expression broadly in wing imaginal discs (Figure 3C). However, expression of *UAS-Pho-RNAi* under the control of *386Y-Gal4*, which drives expression in peptidergic neurons that control wing inflation [21] leads to an inflation phenotype in 51% of flies (n = 136) (Figure 3D). Knock-down of *Spt5* expression by *UAS-RNAi-Spt5* is cell lethal, similarly clones of cells homozygous for null alleles of *Spt5* do not survive (Figure 4), so we were unable to determine if the genetic interaction between *Spt5* and *pho* occurs specifically in peptidergic neurons.

Due to the technical difficulties of studying pupal development, the gene networks that drive eclosion and wing inflation are poorly understood. However, a number of other transcriptional regulators have been implicated in these processes including CREB binding protein (CBP) and the trithorax group protein Ash1 [21]. Our observations demonstrate for the first time that *pho* plays a key role in eclosion, including the process of wing inflation and deflation.

### Pho and Spt5 Bind Overlapping Sites across the Genome

**Figure 5:**
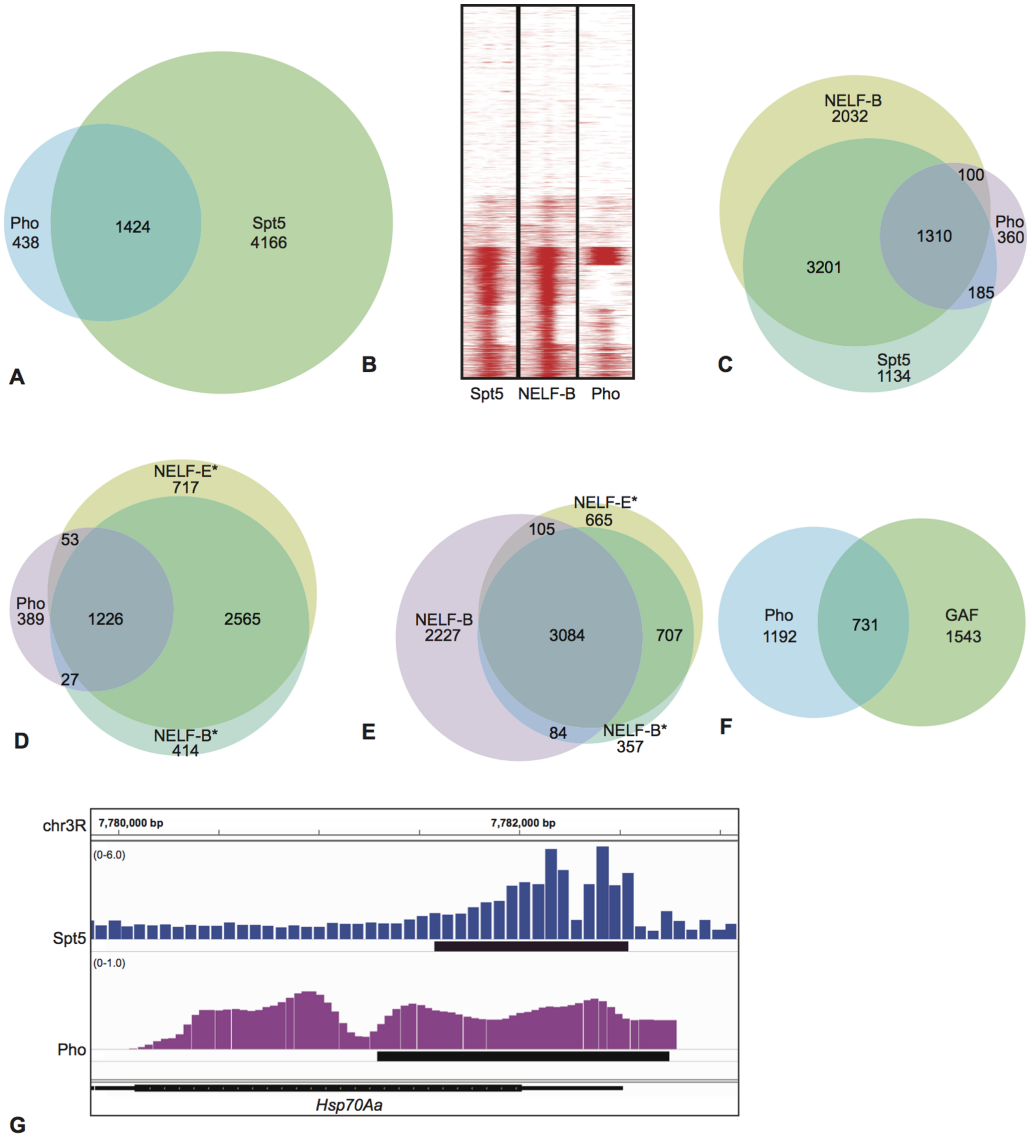
Meta-analysis of ChIP data for Pho [22], Spt5 [25], NELF [25], [31] and GAF [31] binding across the genome of *Drosophila* S2 cells. Asterisks denote the NELF data from [31]. A) Venn diagram showing peaks the overlap of Pho binding with Spt5. B) Heat maps shows peaks of Spt5, NELF-B and Pho binding relative to the TSS (centre of each column) for all coding genes annotated in the genome from the Ensembl database (Release 5.48). Plots show 200 bp up and downstream of the TSS. SEQMINER was used to cluster and visualise the data using the default settings (the 'Kmeans raw' clustering normalization method with 10 expected clusters) [60]. C) Venn diagram showing overlap of Pho and Spt5 peaks with NELF-B binding. D) Overlap of Pho peaks with peaks derived from NELF-B* and NELF-E* datasets. E) Venn diagram showing the overlap between the NELF-B peaks from [25] and NELF-B* and NELF-E* datasets from [31]. Differences in the profiles of NELF binding between these two studies may be due to the use of different antibodies and/or experimental conditions to perform ChIP-chip. F) Overlap between Pho and GAF peaks in S2 cells. Note: the total number of peaks for an individual factor can vary due to the merging of several overlapping peaks into a single peak if multiple peaks of one factor overlap with a single peak of another. G) Overlap of ChIP peaks for Spt5 and Pho binding at the *Hsp70Aa* gene. Note: There are differences in the publicly available track format of Spt5 (bedgraph) and Pho (smoothed wig) data, and differences in the tiling array used for the ChIP-chip experiments (Spt5: Nimblegen Henikoff_Dmel_r52_ChIP tiling design, mean probe length = 53 bp, mean distance between probes = 12 bp; Pho: Affymetrix *Drosophila* v2.0R tiling array, probe length = 25 bp, mean distance between probes = 38 bp).

We performed meta-analysis of Pho and Spt5 data from published chromatin immunoprecipitation (ChIP) experiments in *Drosophila* S2 culture cells to determine if Pho and Spt5 ever co-localize to the same sites in the genome in a given cell type. Peaks of Spt5 binding were identified from data in Gilchrist et al., 2010 and the binding data set for Pho was available as part of the modENCODE project [22]. We identified 5590 binding sites for Spt5 and 1862 for Pho in S2 cells. The vast majority of Pho binding sites (1424/1862; 76%) overlap with Spt5 peaks (Figure 5A), while conversely 25% of Spt5 sites overlap with Pho peaks.

Previous studies have demonstrated that Spt5 binds around the transcription start site (TSS) of genes that recruit RNAP II, and also within the gene bodies of actively transcribed genes [23], [24], [25]. Pho binds target sequences associated with the establishment of PcG complexes, but peaks of binding are also found around the TSS and within the gene body of many genes [19], [26], [27], [28]. Heat maps of Spt5 and Pho binding illustrate that Spt5 and Pho frequently bind overlapping sites at or within 200 bp of the TSS (Figure 5B).

The NELF complex has a well documented role in establishing promoter proximal paused RNAP II in higher eukaryotes including *Drosophila* [7], [29], [30], [31]. Spt5 and NELF co-localize around the TSS of many paused genes in *Drosophila* [25]. We compared the peaks of Pho binding to the peaks of NELF (NELF-B) in S2 cells identified in [25]. The majority of Pho peaks overlapped with peaks of NELF (72%), and 67% of Pho peaks overlap with both NELF and Spt5 (Figure 5C). We also compared peaks of Pho binding to data for NELF-B and NELF-E binding reported in [31]. In this data set, Pho peaks overlap with 74% of NELF-B, 75% of NELF-E and 72% with both NELF-B and NELF-E. Thus the vast majority of Pho binding sites co-localise with binding sites for factors known to regulate pausing. There are many more binding sites for Spt5 and NELF than for Pho in S2 cells, indicating that Pho is not a core component of the machinery regulating transcription elongation, but rather a factor that may influence its activity at a subset of genes.

The ability of Pho to bind Polycomb Response Elements (PREs) in chromatized DNA is augmented by the GAGA factor (GAF; encoded by *Trl*) [32]. Mutations in *pho* and *Trl* interact in genetic assays, but a direct physical interaction between the proteins has not been detected [32], [33], [34]. GAF associates with 39% of the genes that have NELF, and GAF binding is often associated with promoter proximal paused polymerase [31], [35], [36]. We observe that 38% of Pho peaks overlap GAF peaks in S2 cells (Figure 5D). Although GAF may facilitate Pho binding at some sites, its presence is not always necessary for Pho recruitment.

## Discussion

We have detected a physical association of Pho and Spt5 in three different assays; yeast 2-hybrid, GST-pull down and co-immunoprecipitation of tagged proteins. Unfortunately we were unable to co-immunoprecipitate the endogenous proteins as the antibodies generously made available to us against the endogenous proteins were all rabbit polyclonals making co-immunoprecipitation impractical. However, we do find that Spt5 and Pho co-localize to over 1000 peaks of binding in *Drosophila* S2 cells, supporting the model that they can interact directly. We have also detected a genetic interaction between alleles of *pho* and *Spt5* during PcG repression and wing maturation, indicating that they function together in vivo.

Previous studies have generated speculation about a direct interaction between PcG proteins and the transcription elongation complex. In mouse embryonic stem cells (ESCs), there is a well-established link between PcG repression and polymerase pausing at bivalent genes [37], [38], [39]. However, the composition of PcG complexes differs between flies and mice, and YY1 (the mouse orthologue of Pho) is not as commonly associated with PcG complexes as Pho [40]. Thus the observations made in mouse may have limited relevance with respect to our observations in *Drosophila*.

In *Drosophila*, the observation that stalled RNAP II persists in tissues where *Ubx* and *Abd-B* are silenced by the PcG complex lead to the supposition that RNAP II elongation factors “somehow communicate with the PcG-silencing complex” [41]. Others noted that PRC1 preferentially binds to promoters associated with stalled RNAP II in *Drosophila* S2 cells [42].

We have confirmed that there is indeed a direct physical interaction between at least one of the RNAP II elongation factors (Spt5) and one member of the PcG complex (Pho) in *Drosophila.* We have also detected a genetic interaction between the *Spt5^W049^* and *pho^cv^* alleles in vivo. The W049 variant of Spt5 causes ectopic transcription through the P-TEFb checkpoint [11]. Thus, we propose a model in which Spt5 acts together Pho to prevent RNAP II transcribing through the P-TEFb checkpoint to maintain PcG repression. In *Spt5^W049^/+; pho^cv^*/*pho^cv^* flies, the effects of the greatly reduced levels of Pho on PcG repression are exacerbated by a proportion of the remaining Pho interacting with the W049 variant of Spt5 that allows aberrant transcription through the P-TEFb checkpoint.

Pho also functions independently of PcG complexes. One example of this is Pho's function during the recovery from heat shock to repress heat shock gene expression to basal levels [26]. The mechanism to establish recovery from heat shock involves inducing RNAP II to pause at the P-TEFb checkpoint [43]. Observations made by Beisel at al., lead to a speculative model that Pho interacts directly with the RNAP II elongation complex or a remodeling complex [26]. Our observation that Pho interacts with Spt5 supports this model. Mutations in *Spt5* lead to a greatly diminished heat shock response, making it difficult to evaluate the role of Spt5 in heat shock recovery ([11] and BHJ unpublished data). However, Spt5 and Pho co-localize around the TSS of the *Hsp70Aa* gene in S2 cells that have not been heat shocked, consistent with a model in which they interact to establish pausing (Figure 5G).

Spt5 is recruited to RNAP II during the transition from initiation to early elongation [44] and is involved in all transcription irrespective of promoter proximal pausing, thus it is unlikely that Spt5 recruitment is directly dependent on Pho. Pho is a sequence specific DNA binding protein [12]. However, Pho is also found spread across actively transcribed genes, including *hsp70*, where it is involved with re-establishing polymerase pausing after heat shock [26]. It is possible that Spt5 recruits Pho to the gene body of *hsp70,* but depletion of Spt5 is lethal to cells (Figure 4) making it difficult to evaluate the role of Spt5 in Pho recruitment. Alternatively, Pho and Spt5 may be recruited to target genes independently, but interact when recruited in close proximity.

Further studies are required to determine the precise details of how Pho influences polymerase pausing, however our current knowledge of which factors Pho interacts with suggests that it could act by helping to tether the polymerase complex close to TSSs, and/or act by nucleosome remodelling.

It has been proposed that paused polymerase is physically held by factors bound to DNA at promoters, since conditions that disrupt protein-protein and protein-DNA interactions allow transcription to run-on [45]. Furthermore, insertion of spacer sequences into the promoter of the *Hsp70* gene in *Drosophila* does not change the site of transcription initiation but does shift the site where the polymerase pauses [36]. Pho could form part of the tethering complex when it binds close to the TSS and interacts with Spt5. Polymerase pausing is not always associated with repression of gene expression; indeed the majority of NELF target genes show decreased expression after NELF RNAi [30]. Thus Pho and Spt5 may interact to promote pausing at genes where Pho maintains transcriptional activity [13], although we have no formal evidence of this.

Pho/YY1 has also been shown to associate with the INO80 nucleosome remodelling complex in *Drosophila* and mammalian cells [46], [47]. The INO80 complex has been implicated in PcG repression of HOX genes in *Drosophila* [48]. Promoter proximal pausing of RNAP II is linked to a distinctive pattern of nucleosome arrangement around the TSS [25], [49], [50]. GAF has been shown to cooperate with NURF to remodel nucleosomes and increase DNA accessibility at the paused *Hsp70* promoter [51]. However, GAF is not associated with all genes with paused RNAP II. Very little is known about which factors help to establish the nucleosome architecture at genes with paused RNAP II in general, so a role for the Pho/INO80 complex can not yet be excluded.

## Materials and Methods

### Cloning

cDNA clones were obtained for *Spt5* (GH15287), *pho* (RE17954) *Spt4* (LD44495), and *NELF-B* (GH10333) from the *Drosophila* Genomics Resource Center (DGRC).

The region coding for the last 153 amino acids of Spt5 was amplified using KAPA HiFi mastermix (Anachem) from the GH15287 cDNA clone as the template and cloned in frame into pGBKT7 (Clontech) for use as the bait in the yeast-2 hybrid screen. The entire coding regions of *Spt5* and *pho* were also amplified from the cDNA templates and cloned in frame into pGBKT7 and pGADT7 respectively.

To construct plasmids for expression of tagged proteins in *Drosophila* cell culture, the coding regions of these cDNAs were amplified and appropriate primers and cloned into pENTR-D using a pENTR(TM)/D-TOPO(R) Cloning Kit (Life Technologies - Invtrogen Division). The coding regions were subsequently cloned into pAMW (N-6×Myc tag) and pAFW (N-FLAG tag) (*Drosophila* Gateway Vector Collection distributed by the DGRC) using the Gateway LR Clonase II kit (Life Technologies - Invtrogen Division). The Myc-tagged GFP clone was a gift from Nic Tapon. All primer sequences used for cloning are available on request.

### Yeast 2-hybrid Screen

The yeast 2-hybrid screen was performed using the Matchmaker Gold Yeast Two-Hybrid System from Clontech following the manufacturer's protocols. Approximately 6.4 million colonies of the Clontech Universal *Drosophila* (Normalized) Mate & Plate Library were screened with the C-terminal 153 amino acids of *Drosophila* Spt5 cloned in frame into pGBKT7. Three independent clones expressing in frame sequences of *pho* were recovered from the screen. These clones did not activate expression of reporter genes in the yeast in the absence of Spt5.

### In Vitro Protein-Protein Interactions

GST pull-down experiments were performed as described previously [52]. pGADT7-pho was translated using the TNT T7 Quick Coupled Transcription/Translation System (Promega). Co-immunoprecipitations were performed essentially as described by [53]. S2R+ cells were grown in Scheider's insect medium (Sigma) supplemented with 10% FCS and 1% Penicillin/Streptomycin, were transfected using Effectene (Qiagen) according to the manufacturer’s instructions. Myc-tagged proteins were detected using anti-Myc Antibody (A-14): sc-789 rabbit polyclonal IgG from Santa Cruz and FLAG-tagged proteins using Monoclonal ANTI-FLAG(R) M2 antibody produced in mouse (Sigma). Western blots were visualized using the ECL plus kit (GE Healthcare) and Kodak(R) BioMax(TM) MR film.

### Drosophila Strains

The *pho^cv^/ci^D^* stock was a gift from Ana Busturia and *P{neoFRT}82B cu^1^ sr^1^ NELF-A^KG^/TM3* flies [18] a gift from Peter Gergen. The *Spt5^W049^* stock has been described previously [11]. *y^1^ w^1118^; P{lacW}M64 P{lacW}G38 P{lacW}J29 P{EP}wech^EP813^ P{lacW}K61 Spt5^MGE^* ^−*3*^ */SM1* were obtained from the Bloomington *Drosophila* Stock Center. *Spt5^MGE^* ^−*3*^ was recombined on to *FRT42B* to clean up the chromosome and to facilitate clonal analysis. The *765-Gal4* driver line has been previously described in [54] and the *386Y-Gal4* and *da-GAL4* driver lines were obtained from the Bloomington *Drosophila* Stock Center. The *UAS-RNAi-pho* flies used in this study were *w^1118^; P{GD1509}v39529* from the Vienna Drosophila RNAi Center (VDRC) [55]. Somatic clone induction, immunohistochemistry and germ-line clone analysis were carried out as described previously [56]. The *UAS-RNAi-Spt5* flies used in this study were *P{KK101304}VIE-260B* from the Vienna Drosophila RNAi Center (VDRC) [55].

### Analysis of Published ChIP-chip Data

Peaks were identified with Ringo [57] using the default parameters and a threshold cutoff of 1.5 for the ratio of Spt5 to input binding on the Nimblegen *Drosophila* tiling array [25] (GEO accession number GSE20472). Similarly peaks were called for NELF-B [25] (GEO accession number GSE20472) with Ringo using the default parameters. The average probe signals were smoothed, and the 95^th^ quantile of the log2 immunoprecipitated/input ratio was used as the cutoff value for the detection of peaks. For all other ChIP data, peaks were taken from the published literature. The data for Pho was from modENCODE [22] (http://www.modencode.org), NELF-B*, NELF-E* and GAF binding was from [[31] ArrayExpress (www.ebi.ac.uk/arrayexpress/) under accession number E-MEXP-1547]. Identification of overlapping peaks was determined by the ChIPpeakAnno package [58] and if multiple peaks from one factor overlapped with a single peak from another factor, then the peaks would be merged in the calculation of the number of overlapping peaks. Venn diagrams were generated with venneuler [59]. Heat maps of binding sites relative to TSS were generated with the SeqMiner program [60].
